# ARTP Mutagenesis of *Phanerochaete chrysosporium* BZ103 to Enhance Laccase Activity and Transcriptomic Analysis of the Mutants

**DOI:** 10.4014/jmb.2502.02014

**Published:** 2025-06-12

**Authors:** Shuxin Liu, Ting Liu, Jiale Chen, Haizhou Xu, Dianpeng Zhang, Hongwei Fu, Hongxin Zhao

**Affiliations:** 1 Zhejiang Province Key Laboratory of Plant Secondary Metabolism and Regulation, College of Life Sciences and Medicine, Zhejiang Sci-Tech University, Hangzhou 310018, P.R. China; 2 Wenzhou Rural Revitalization and Development Center, Wenzhou 325019, P.R. China; 3 Institute of Plant Protection, Beijing Academy of Agriculture and Forestry Sciences, Beijing 100097, P.R. China

**Keywords:** White-rot fungi, laccase, ARTP, transcriptomics, multicopper oxidase genes

## Abstract

Laccases (EC1.10.3.2) are copper-containing polyphenol oxidases with broad applications in wastewater decolorization and fabric bleaching. However, the current levels of laccase production by existing strains are limited, restricting their practical applications. Further research is needed to improve laccase production and understand their secretion mechanisms in white-rot fungi. In this study, atmospheric and room temperature plasma (ARTP) mutagenesis was performed on the white-rot fungus *Phanerochaete chrysosporium* BZ103, resulting in the selection of two laccase-hyperproducing mutants, Tx426 and Wx089, with 45 and 30% higher titers, respectively. Transcriptome sequencing revealed 272 upregulated and 226 downregulated genes in the mutant strain Tx426 compared to the original strain (CK), as well as 434 upregulated and 228 downregulated genes in Wx089. Notably, the multicopper oxidase genes *mco3B* and *mco2A* were significantly upregulated in the laccase-hyperproducing mutants, with expression levels increased by 2.03 and 2.33 times for Tx426 vs. CK, as well as by 1.14 and 1.33 times for Wx089 vs. CK. Based on the transcriptome analysis results, the multicopper oxidase genes *mco3B* and *mco2A* were significantly up-regulated in the high-yielding laccase mutant strain. Although there is no direct evidence that they are mutated, the significant changes in gene expression are closely related to the enhancement of laccase activity. This study preliminarily analyzed the possible factors related to the synthesis and regulation of laccase in the mutants of *P. chrysosporium*. The significantly upregulated multicopper oxidase genes *mco3B* and *mco2A* in the mutants may play important roles, but the specific mechanism requires further in - depth research.

## Introduction

Laccases (EC1.10.3.2) are multicopper oxidases with great potential for bioremediation and decolorization[1] . As such, they are widely used to degrade a variety of xenobiotic compounds, including synthetic dyes [2], polycyclic aromatic hydrocarbons [3], pesticides, drugs such as diclofenac, and endocrine disruptors [4, 5].

Most laccases used today are derived from white-rot fungi such as *Phanerochaete chrysosporium* [6, 7], which was also utilized in this study. Despite the identification of specific genes related to laccase expression and secretion [8, 9], the lack of studies on potential molecular and metabolic mechanisms associated with laccase activity, combined with the disadvantages of white-rot fungi such as long fermentation period[10], low laccase yield[11], and instability[12], has hampered further strain engineering and process optimization in an industrial setting. Therefore, it is crucial to select and cultivate superior strains as well as to investigate potential metabolic pathways for enhancing laccase activity[13].

ARTP is a powerful technology used to induce genetic mutations in microorganisms for the purpose of selecting improved mutants [14, 15]. It utilizes the unique properties of atmospheric pressure plasma sources to generate a wide range of genetic changes in microorganisms, which can then be screened for desirable traits [16, 17]. This technology has significantly increased the ability to develop improved microbial strains for various biotechnology applications, leading to a wide range of industrial applications, such as these production of antibiotics, enzymes, biofuels, and other high-value-added products [18-20]. Therefore, this study mined the relevant molecular mechanisms for enhancing laccase activity through ARTP mutagenesis combined with transcriptome analysis. As a mature research tool, transcriptome analysis provides an effective technical tool for comprehensively resolving mutant gene expression levels in microbial breeding [21, 22]. Transcriptomics aids in validating gene function by linking changes of gene expression to the phenotypes of mutants [23, 24]. A recent study screened laccase hyperproducing mutant strains generated through ARTP mutagenesis, and explored genes that may enhance laccase activity by transcriptome analysis on the Illumina Hi-seq sequencing platform. Combined with these previous findings, the results of this study help us understand the genetic mechanism of laccase activity regulation and provide a basis for future genetic modification and directed evolution of laccase-producing industrial strains.

## Materials and Methods

### Strain and Growth Condition

The newly-isolated white-rot fungal strain BZ103, capable of effective decolorization, was provided by the Microbiology Laboratory of Beijing Academy of Agricultural Sciences, Beijing, China.

The optimized medium for BZ103 to maximize laccase production, which was determined via Box-Behnken design, comprised glucose 10 g/l, peptone 0.206 g/l, potassium dihydrogen phosphate 2 g/l, magnesium sulfate 0.5 g/l, iron sulfate heptahydrate 0.115 g/l, calcium chloride 0.1 g/l, zinc sulfate heptahydrate 0.089 g/l, vitamin B1 0.001 g/l, manganese sulfate monohydrate 0.54 mmol/l, Copper sulfate pentahydrate 0.11 mmol/l, with a C/N ratio of 91.6 and a pH of 4.0. This optimized medium was used for the subsequent cultivation of the white-rot fungus BZ103, with an inoculum size of 8.4%.

### Quantitative Assessment of the Dye Degradation Rate

Seven common dyes (azo dyes: Congo red and Evans blue; triphenylmethane dye: crystal violet; phenothiazine dye: azure A; indigo dye: indigo carmine) were added to the optimized culture medium at a final concentration of 2 mg/l, followed by inoculation with BZ103. The wavelengths were set at 200-800 nm, and the maximum absorption wavelengths of each dye were determined by full-wavelength scanning with an ultraviolet spectrophotometer (MD1000, Thmorgan, China), and the decolorization efficiency of the laccase produced by BZ103 for each dye was calculated.

The formula for calculating the decolorization efficiency of the laccase produced by BZ103 towards dyes was as follows:

$$R=\frac{A_{0}-A_{1}}{A_{0}}\times 100\%$$

where R represents the decolorization rate of the dye, while A0 and A1 represent the absorbance values of the solution before and after the decolorization reaction, respectively.

### Laccase Extraction and Activity Assay

BZ103 cultures were centrifuged at 4,500 rpm for 5 min to separate the supernatant, which was further centrifuged at 8,000 rpm for 10 min at 4°C to obtain a clarified supernatant, designated as the crude laccase enzyme solution. The reaction mixture comprised 2.7 ml of 50 mM acetate buffer (pH 5.0), 0.1 ml of 1 mM ABTS (2,2'-Azino-bis (3-ethylbenzothiazoline-6-sulfonic acid)), 0.1 ml of 20 mM H_2_O_2_, and 0.1 ml of the crude enzyme solution. The reaction was carried out at 30°C for 24 h. The absorbance at 420 nm (A_420_) was measured to determine the enzyme activity. One unit of enzyme activity was defined as the amount of enzyme required to catalyze the oxidation of 1 μmol of ABTS per minute. The molar absorptivity (ε_420_) at 420 nm was 3.6 × 10^4^ L/(mol·cm).

The formula for determining laccase activity was as follows:

$$\text{Laccase activity }\left(\text{U/L}\right)=\frac{\Delta A_{420}\times V_{A}\times n}{\Delta t\times V_{B}\times \varepsilon \times 10^{-6}}$$

Where ΔA_420_ is the absorbance change at 420 nm, V_A_ is the total volume of the reaction system (ml), n is the dilution ratio, Dt is the reaction time (min), V_B_ is the volume of diluted enzyme solution (ml) in the reaction system, ε is the extinction coefficient L/(mol·cm), 10^-6^ is the conversion ratio of mol to μmol.

### Quantification of Genes Related to Laccase Expression

The NCBI database was used to search for several related multicopper oxidase sequences from *P. chrysosporium*, and in conjunction with existing studies, it was determined that the multicopper oxidase genes under study mainly include *mco3B* and *mco2A*, and it was shown that these genes may be closely related to laccase activity [25]. The resulting fasta file was imported into MEGA (MEGA11) software for homologous sequence comparison, and corresponding sequences were selected for primer design to investigate their presence in the tested strains. In the primer design section, the specific information of the genes on which the primer design was based, the primer sequences (*mco3B*-F: 5'-TTACCCTTGGCGAGTTCTCTCTG-3'; *mco3B*-R: 5'-ACCTGTATCGAATCCTCCGTGA-3'), and the software for the primer design (Premier v5.0). The amplicons were recovered by excision of the agarose gel containing the corresponding bands, followed by confirmation via Sanger sequencing. After obtaining the corresponding sequence, the next step was to analyze the expression of the multicopper oxidase gene in the presence of seven different dyes by fluorescence quantitative PCR. In the experiments, the β-actin gene was selected as the internal reference gene and relative expression was calculated using the 2-ΔΔCt method as follows:

ΔΔCt = ΔCt (Treatment) - ΔCt (CK)

ΔCt = Ct (Target gene) - Ct (Internal gene)

BZ103 mycelia cultured with various dyes were collected and ground into a powder in liquid nitrogen. Total RNA was then extracted using the Fungal Total RNA Isolation Kit (Sangon Biotech Co., Ltd., China). Subsequently, cDNA was synthesized using the ReverTra Ace qPCR RT Master Mix Kit with gDNA Remover (Toyobo Co., Ltd., Japan). The relative expression levels of the multicopper oxidase gene in the mycelia cultured with the seven dyes were determined using the SYBR Green Realtime PCR Master Mix Kit (Vazyme, China).

### Screening of High-Laccase-Producing Mutants of BZ103

BZ103 was cultivated on PDA medium under low light conditions at 28°C for three days. Spores in the exponential growth phase were collected and exposed to the ARTP mutagenesis system (ARPT-IIS, Wuxi Tmaxtree Biotechnology Co., Ltd., China) [26]. After ARTP treatment for durations of 0 s, 30 s, 60 s, 90 s, 120 s, 140 s, 160 s, and 180 s, respectively, the samples were diluted and plated onto PDA solid medium. The plates were then incubated at 30°C for 18 h. The optimal mutagenesis time was determined through colony counting, and a lethality curve was plotted and calculated based using the equation

$$\text{Lethality rate}\left(\%\right)=\frac{T-A}{T}\times 100\%$$

where *T* is the total colony count of the control sample without ARTP treatment, and *A* is that of the test sample after ARTP treatment. The optimal mutagenesis conditions were determined according to the curve, to achieve a lethality rate of approximately 90%.

Fungi produce laccase that can oxidize and decompose guaiacol, generating a reddish-brown compound [27]. By adding guaiacol to the medium, color changes can be used to indirectly assess laccase production [28]. After the optimal mutagenesis time, the spore suspension treated with ARTP was plated onto a PDA plate and incubated at 30°C for 18 h. Mutants with fast and robust mycelial growth were selected and transferred onto PDA medium containing 1% guaiacol, followed by incubation at 30°C for 6 days. During the initial screening, mutants with darker coloration were chosen, and their laccase activity was measured for further screening.

### Selection of Stable Mutants

Strains with laccase production more than 10% higher than BZ103 were subjected to fifteen rounds of subculture. The laccase production of both the original strain and the mutant strains was determined in three replicate fermentations. If the laccase activity of the mutagenized strain consistently increased over five generations and remained significantly higher than that of BZ103, it was deemed genetically stable.

### Transcriptome Sequencing

Wild-type *P. chrysosporium* BZ103 (CK) and two mutants (Tx426 and Wx089) were chosen for transcriptome analysis. First, the three strains were streaked onto a PDA solid plate and cultured for 96 h, after which the mycelia were transferred into PDB liquid medium, followed by culture at 30°C and 200 rpm for 72 h. The resulting seed culture was used to inoculate the optimized medium for laccase production at an 8.4% inoculation ratio, followed by shake-flask cultivation at 30°C and 200 rpm for 96 h. After centrifugation to harvest the mycelia, the total RNA was extracted using a Total RNA Extraction System (Takara, Japan), after which the mRNA was purified and fragmented to approximately 300 bp. These fragments were used as templates to synthesize the cDNA. After purification, samples were sent to Meiji Biological Co., Ltd. Shanghai headquarters, for Illumina HiSeq transcriptome sequencing (PE library, Read 2×150 bp) [29]. The raw sequencing data have been uploaded to the NCBI's Sequence Read Archive (SRA) database, and the corresponding accession number (BZ103 accession number: SRR27430906; SRR27430905; SRR27430904, Tx426 accession number: SRR27430901; SRR27430902; SRR27430903, Wx089 accession number: SRR27430898; SRR27430899; SRR27430900) and date (access date: 2024-01-05) has been obtained.

### Data Assembly and Functional Analysis of DEGs

An Illumina PE library suitable for 2×150 bp sequencing was constructed. DESeq2 was utilized to quantify gene expression and analyze the expression levels of various genes across samples. The experiments were all set up with three sets of replicates. Only genes meeting the criteria |log_2_ fold change| ≥ 1 and *P* < 0.05 were identified as differentially expressed genes (DEGs), which were then analyzed and aligned with NR, EggNOG, GO, as well as KEGG to obtain annotation information from each database. Statistical analysis was conducted on the annotation information obtained from each database.

### Statistical Analysis

The one-way ANOVA was performed in SPSS24 to analyze the dye degradation, laccase activity and genetic stability.

## Results and Discussion

### Decolorization of Dyes by BZ103

**Fig. 1:**
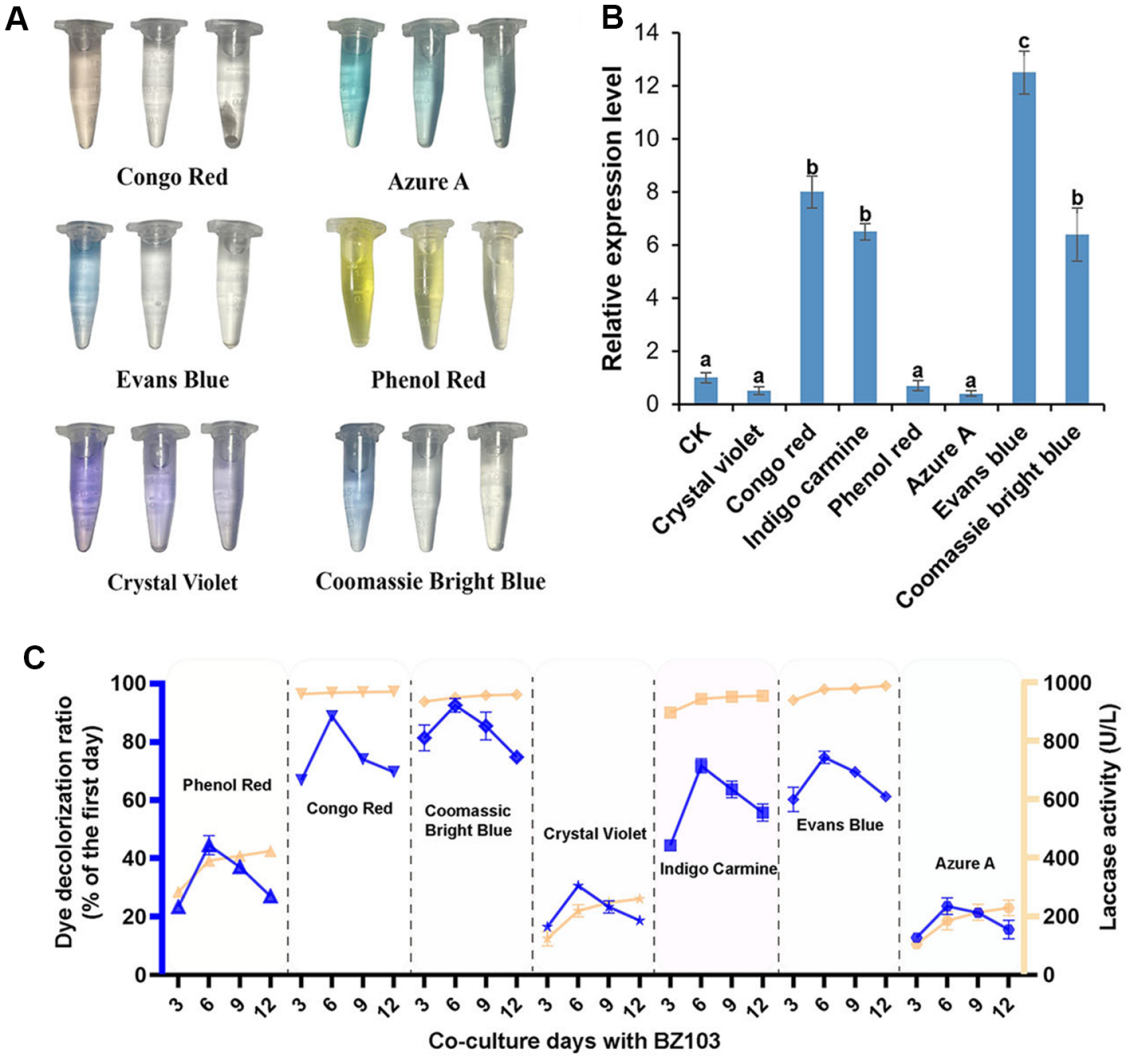
Identification of dye decolorization effect by laccase produced by BZ103. (**A**) Visual representation of dye decolorization after co-culturing six synthetic dyes (Congo Red, Azure A, Evans blue, Phenol Red, Crystal Violet, Coomassie Bright Blue) with BZ103 for 0, 3, and 6 days, respectively. (**B**) Changes in dye decolorization and corresponding laccase activity when seven synthetic dyes (Congo Red, Azure A, Evans blue, Phenol Red, Crystal Violet, Coomassie Bright Blue, Indigo Carmine) were co-cultured with BZ103 for 3, 6, 9, and 12 days, respectively (The data in red font is the maximum absorption wavelength of the dyes). (**C**) RT-PCR identification of the relative expression of multicopper oxidase genes in BZ103 after coculturing with seven synthetic dyes for 6 days.

Laccase, a polyphenol oxidase that catalyzes or oxidizes various substances with only water as a by-product [30], has a strong potential for application as it plays a role in the degradation of various organic pollutants (phenolic and non-phenolic, etc.) and in the decolorization of dyestuffs [31]. The experimental results showed that seven synthetic dyes were incubated with BZ103 mycelia for different durations, resulting in varying degrees of decolorization. Among them, Congo red, Coomassie brilliant blue, indigo carmine, and Evans blue exhibited the best degradation effects, with decolorization rates approaching 100% after 12 days of fermentation (Table 1), which suggests that laccase may have high application value in dealing with these specific dye contaminations. However, the decolorization rates of phenol red, crystal violet, and azure A remained below 50% on the 12th day (Fig. 1A and 1B). This may be related to laccasés low affinity for these dyes or the difficulty of their structure in being effectively degraded by laccase [32]. Next, the correlation between dye decolorization rates and laccase activity was determined. Compared to the control group without added dyes, the addition of Congo red, Coomassie brilliant blue, indigo carmine, and Evans blue significantly increased laccase activity (*p* < 0.05). By contrast, azure A, phenol red, and crystal violet had inhibitory effects on laccase activity to varying degrees (Table 2 and Fig. 1B). This suggests that there are differences in the induction or inhibition of laccase by different dyes, which may be related to the chemical structure of the dyes and the substrate specificity of laccase [33]. RT-qPCR analysis revealed that Congo red, indigo carmine, Evans blue, and Coomassie brilliant blue significantly upregulated the expression of the multicopper oxidase gene. In the presence of Evans blue, the upregulation of the gene was the most significant, approximately 12 times that of the control group. By contrast, when crystal violet, phenol red, and azure A were present, the expression level of this gene was significantly reduced, with the most pronounced inhibition observed in the presence of azure A, which was only one-sixth of the control group. With the extension of the incubation time, the amount of laccase secreted by *Phanerochaete chrysosporium* BZ103 gradually increased, and the degradation of the dye was continuously enhanced, increasing the decolorization rate. At the early stage of culture, the strain needed some time to adapt to the environment and initiate laccase synthesis, so the decolorization rate was relatively low at 3 days. And at 6, 9 and 12 days, the laccase production reached a high level and the degradation of dyes was more significant. The changes in the expression level of the multicopper oxidase gene were consistent with the trend of enzyme activity changes, suggesting that the protein encoded by this gene is the primary laccase of the strain (Fig. 1C). To summarize the above, laccase shows great potential in treating specific dye contaminants, but its efficiency is affected by the type of dye. Future studies could further explore how to improve the efficiency of laccase in the treatment of difficult-to-biodegrade dyes through genetic engineering or optimization of culture conditions [34] and how to utilize this selectivity of laccase for the development of more efficient dye decolorization and organic pollutant degradation technologies.

### Screening of High-Yield Laccase-Overproducing Mutants

**Fig. 2:**
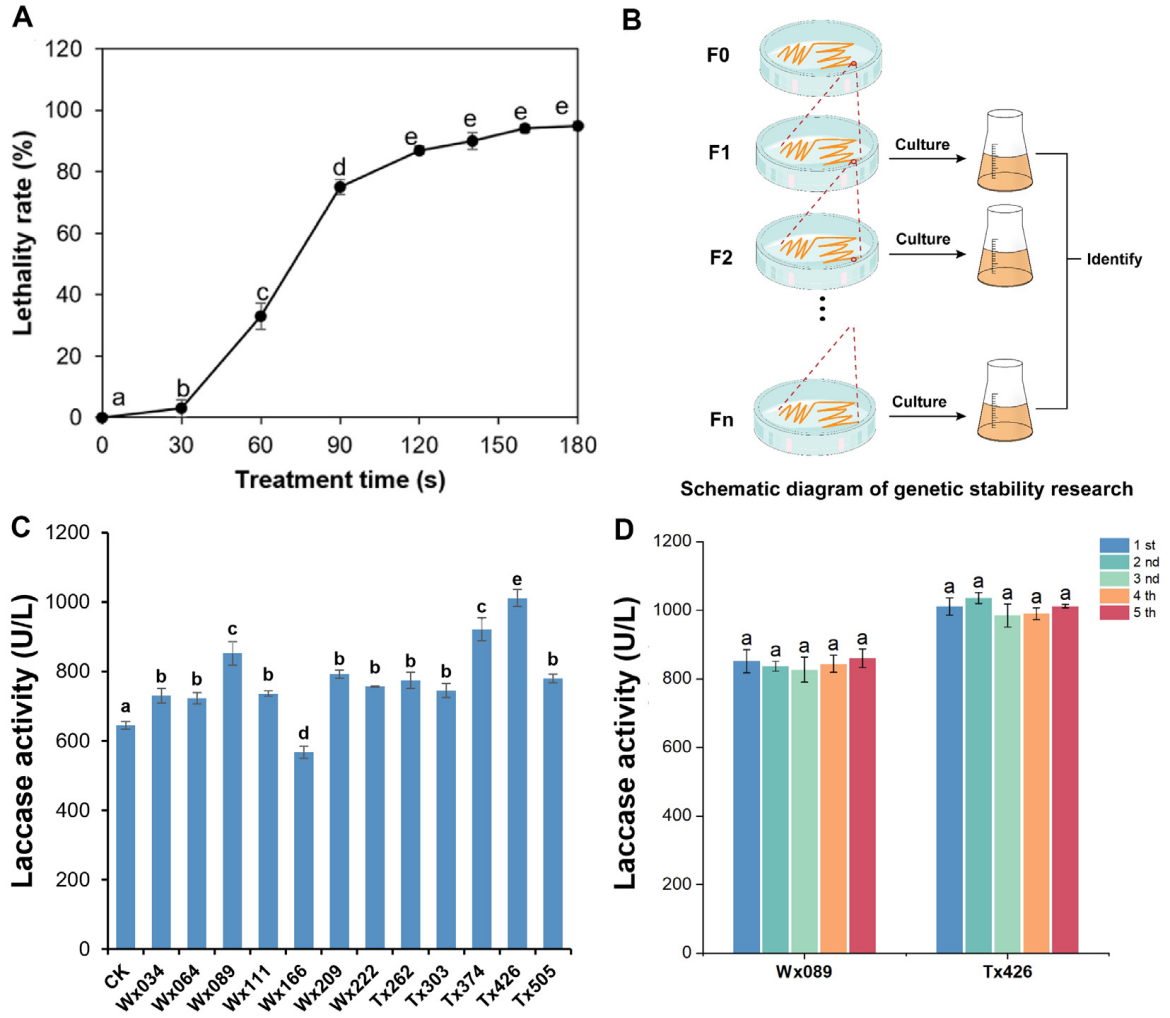
Screening of Laccase-Hyperproducing Mutant Strains. (**A**) Effect of ARTP treatment duration on the lethality rate of BZ103. (**B**) Schematic diagram of stability screening for mutant strains. (**C**) Genetic stability assessment, where the enzyme activity of the initially screened high-yield mutant strain is measured after continuous culturing for 5 generations. (**D**) Enzyme activity measurement of the selected optimal high-yield mutant strain over 5 consecutive generations for laccase production.

The mutation frequency of atmospheric and room temperature plasma (ARTP) mutagenesis was closely related to the mortality of the strain. Achieving a lethality rate of approximately 90% is generally considered the best option for generating sufficient positive mutations (Yang *et al*. 2019). To assess the optimal ARTP treatment time, the mortality curve was plotted (Fig. 2A). Treatment times with mortalities above 80% (120s, 140s) were selected for mutagenesis of BZ103. This result highlights the potential of ARTP technology in enhancing the target enzymés activity while strengthening the rigor of the experimental results through a multi-generation genetic stability test.

Utilizing the characteristic guaiacol oxidation by laccase, which produces a reddish-brown substance, 524 strains obtained through mutagenesis were reduced to only 96 high-laccase-producing strains through initial screening. These newly mutated strains were subjected to shake flask re-screening, resulting in 12 strains with increases of laccase activity by more than 10%. Genetic stability tests were then conducted on these 12 strains by subculturing them five times and measuring the enzyme activity of each generation. Among them, Wx089, Wx222, Tx374, and Tx426 showed an increase of enzyme production above 10% (Fig. 2C), and the enzyme production of Wx089 and Tx426 increased significantly, and the relative standard deviation (RSD) of their laccase production was calculated. The results showed that RSD of Wx089 was 1.89%, while that of Tx426 was 2.12%. Hence, both were lower than 5%, indicating that the laccase yield has good genetic stability in the short term. The laccase activities of these strains were respectively 1.3 and 1.45 times that of the original strain (Fig. 2D). This study highlights the significant impact of ARTP technology on enhancing the enzyme production capabilities of microorganisms. It also confirms the stability of these improved strains in practical applications through thorough genetic stability testing. These findings provide a strong foundation for future strain optimization and enzyme engineering research.

### Differential Expression Analysis

**Fig. 3:**
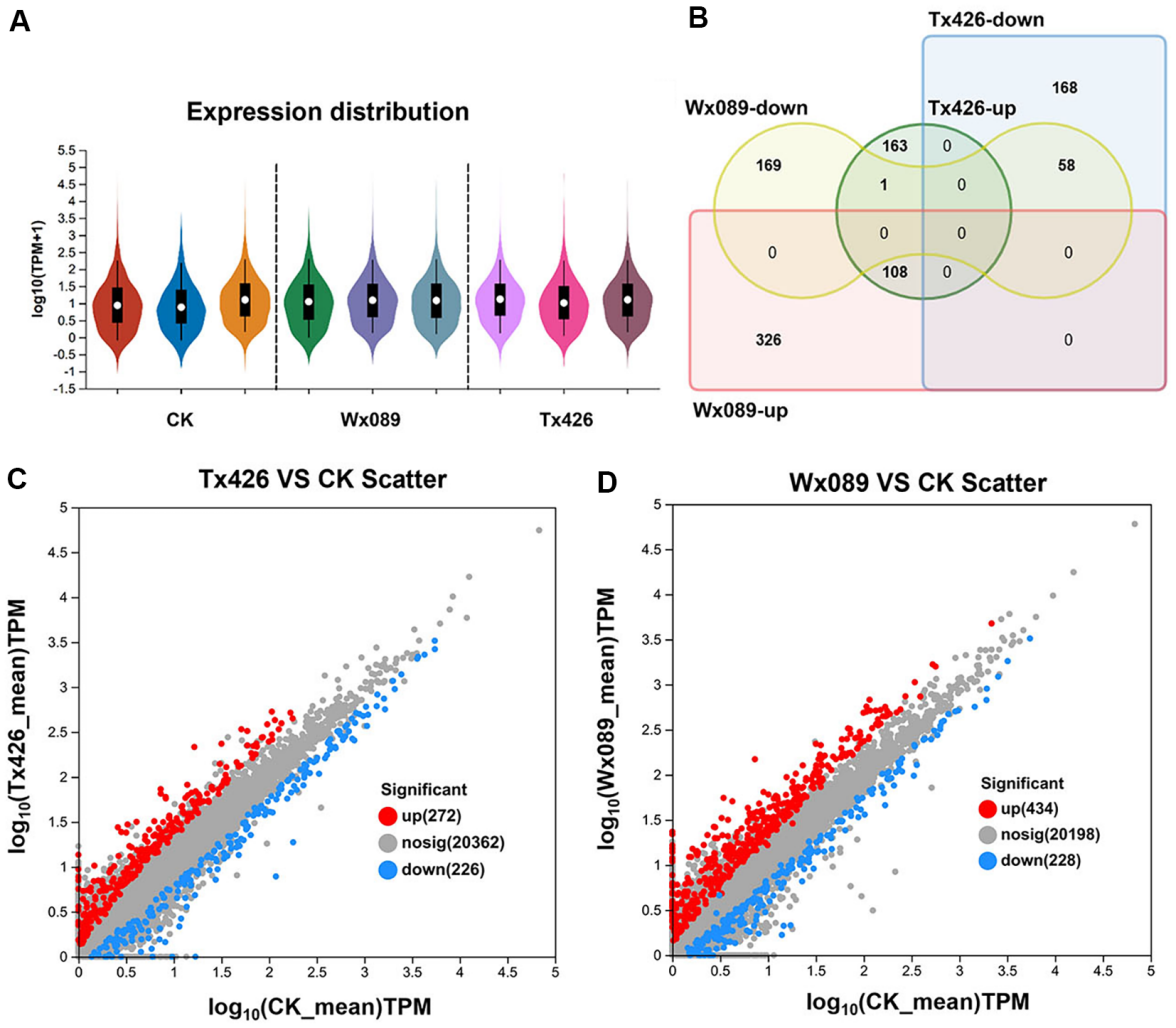
Transcriptome Differential Analysis Between High-Yield Mutant Strains Wx089 and Tx426, and BZ103. (**A**) Schematic representation of gene expression levels in laccase high-yield mutant strains and the control group. (**B**) Venn diagram illustrating the DEGs (differentially expressed genes) between BZ103 and laccase high-yield mutants Wx089 and Tx426 (circles of different colors represent different gene sets, and the numbers represent the number of shared or unique genes between different gene sets).Red: Wx089 significantly upgraded; Yellow: Wx089 significantly downgraded;Green: Tx426 significantly upgraded; Blue: Tx426 significantly downgraded?Red and green overlap means: Wx089 and Tx426 are jointly upregulated; Yellow and green overlap means: Wx089 and Tx426 are synchronized downward. (**C**) Scatter plot of DEGs between BZ103 and laccase high-yield mutant Tx426 (red dots represent significantly upregulated genes, blue dots represent significantly down regulated genes, and gray dots represent genes with insignificant differences in expression). (**D**) Scatter plot of DEGs between BZ103 and laccase high-yield mutant Wx089.

Read counts have inherent biases when reflecting gene expression levels [35]. To mitigate these biases, read counts were normalized using the TPM (transcripts per million) method. The TPM method makes expression levels comparable across samples by adjusting the read counts for each sample to reflect the ratio per million transcripts [36]. Subsequently, quantitative analysis of gene expression levels was conducted using RSEM (RNA-Seq by expectation- maximization) software, with TPM as the quantitative metric based on the number of transcripts. RSEM is an advanced quantitative tool for RNA sequencing that estimates the expression levels of genes and transcripts through an expectation-maximization algorithm to provide more accurate expression estimates [37]. In this study, only genes with | log2 FC| ≥ 1 and *p*-adjust < 0.05 were identified as differentially expressed genes (DEGs), and this criterion was used to screen and analyze the differences in gene expression between the *Phanerochaete chrysosporium* BZ103 and its mutant strains after ARTP mutagenesis treatment. The outcomes demonstrated an elevation in gene expression levels in the mutagenized strains compared to the parental strain (Fig. 3A). To explore the molecular response to ARTP mutagenesis in BZ103, a comparative analysis was conducted between the high-yield mutant strain Tx426 and the wild type, revealing that 272 genes were significantly up-, and 226 downregulated (*p* < 0.05) (Fig. 3C). In the second high-yield mutant strain Wx089, 434 genes were significantly up-, and 228 were downregulated (*p* < 0.05) (Fig. 3D).

Venn diagram analysis of the differentially expressed genes (DEGs)between the two high-yield mutant strains, Wx089 and Tx426, showed that 108 DEGs were simultaneously upregulated in both mutant strains, while 58 were downregulated (Fig. 3B). This demonstrates that the number of differential genes significantly increased after ARTP mutagenesis, and some genes exhibited similar expression patterns in both high-yield mutants. These findings provide an important perspective for understanding the effects of ARTP mutagenesis on gene expression and lay the foundation for subsequent studies of molecular mechanisms.

### GO Classification Statistics and Enrichment Analysis of DEGs

**Fig. 4:**
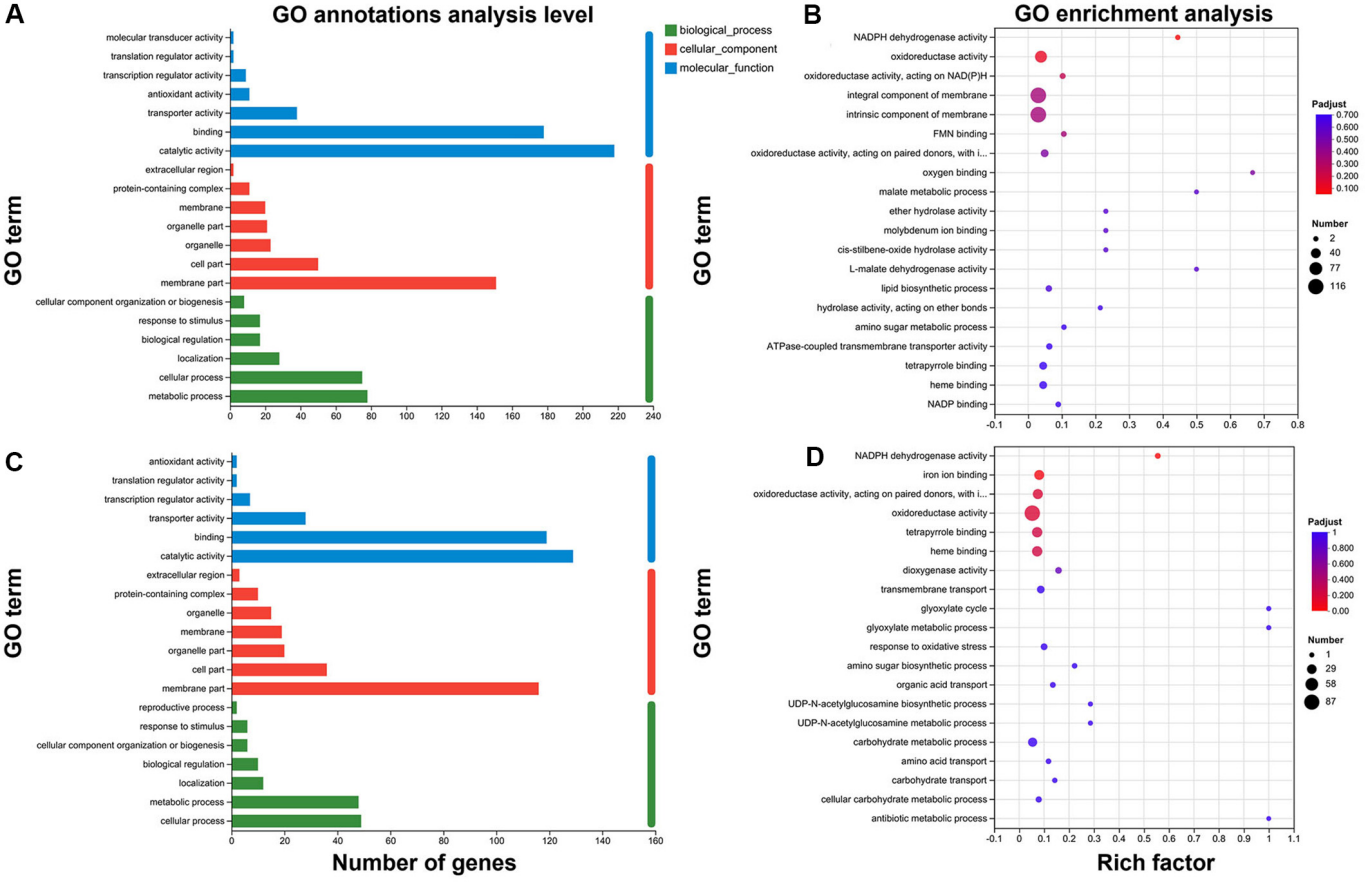
GO Functional & Enrichment analysis of DEGS. (**A**) GO functional analysis of DEGs (BZ103 VS Tx426). (**B**) GO functional analysis of DEGs (BZ103 VS Wx089). (**C**) GO enrichment analysis of DEGs (BZ103 VS Tx426). (**D**) GO enrichment analysis of DEGs (BZ103 VS Wx089).

The gene ontology (GO) database is an international system for standardized classification of the functions of genes and gene products across different species. It classifies genes into three main categories: biological process (BP), cellular component (CC), and molecular function (MF). The classification of DEGs based on the GO database indicate that the DEGs between both high-yield mutant strains Wx089 and Tx426 compared to the wild type were primarily concentrated in the cellular component and molecular function categories. These genes are mainly related to membrane part transporter activity, binding, and catalytic activity (Fig. 4A and 4B). These genes play crucial roles in cellular metabolism and material transport. Therefore, the increased laccase production in the high-yielding mutant strains Wx089 and Tx426 may be related to changes in their expression. Functional significance enrichment analysis based on GO enrichment analysis (with P-adjusted < 0.05) revealed a significant enrichment of oxidase genes (Fig. 4C and 4D). Oxidative enzymes have a variety of functions in living organisms, including participation in redox reactions and substance metabolism[38]. It stands to reason that the enrichment of oxidase genes was related to the high laccase production in the mutants derived from BZ103.

### Functional Analysis and Validation based on the KEGG Database

**Fig. 5:**
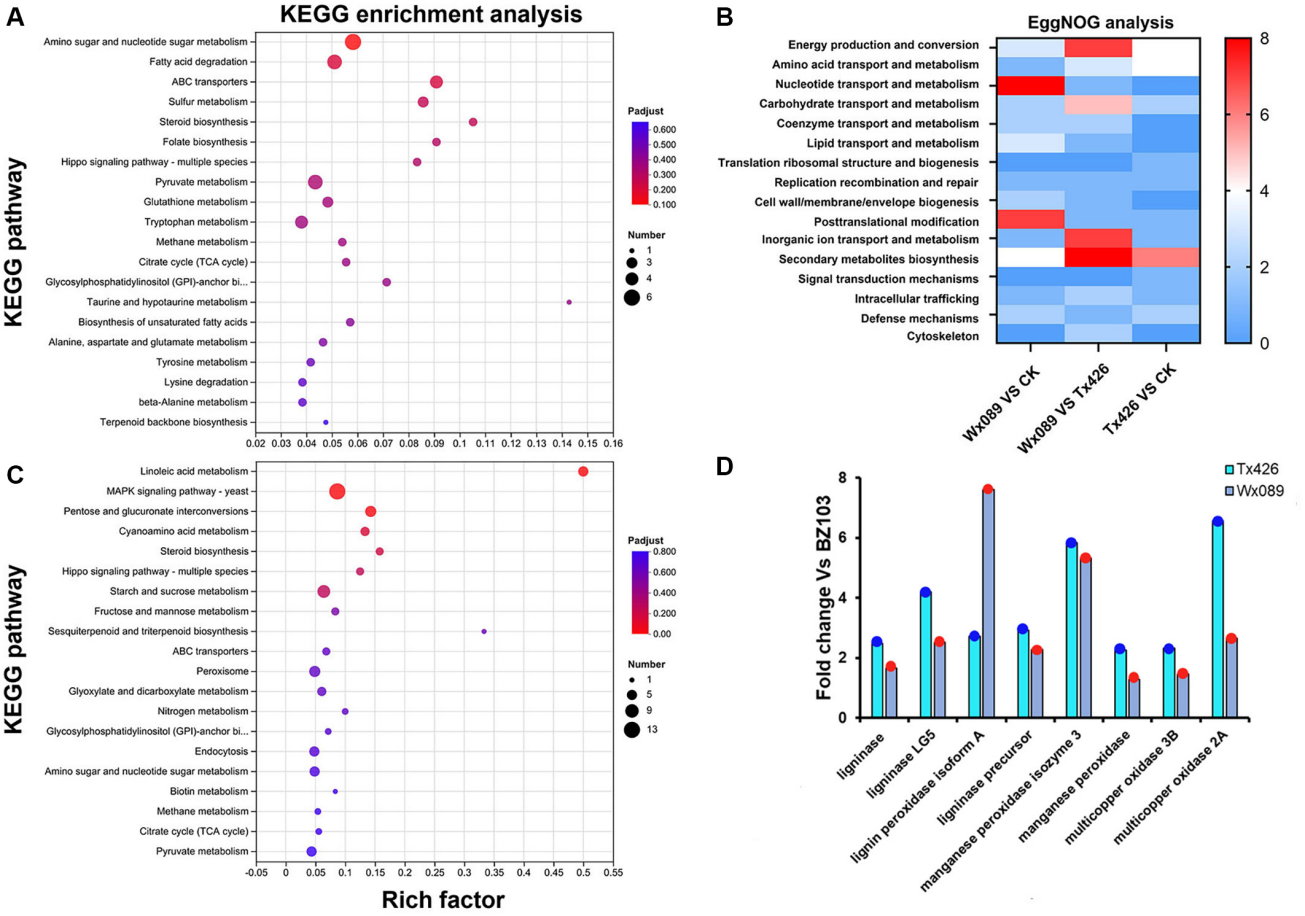
Functional Analysis and validation of candidate genes. (**A**) KEGG enrichment pathway analysis of DEGs (Tx426 VS CK). (**B**) KEGG enrichment pathway analysis of DEGs (Wx089 VS CK). (**C**) EggNOG analysis of DEGs (Wx089 VS CK, Wx089 VS Tx426, Tx426 VS CK). (**D**) RT-qPCR Detection of Expression of Related Candidate Genes.

Based on the KEGG database, the DEGs were classified according to the pathways they participate in or the functions they perform. The main categories of DEGs included carbohydrate metabolism, amino acid metabolism, lipid metabolism, energy metabolism, membrane transport, and signal transduction (Fig. 5A and 5B). Using functional annotations from the EggNOG database, it was found that after mutagenesis, the functions of the DEGs between the mutant strains and the wild type mainly encompassed energy production and conversion, biosynthesis of secondary metabolites, transport and catabolism, carbohydrate transport and metabolism, as well as inorganic ion transport and metabolism (Fig. 5C). Remarkably, these changes in functional categories are closely linked to the adjustment of metabolic pathways, reflecting the potential impact of mutagenic treatments on metabolic networks.

Using the NR database, five multicopper oxidase genes of *P. chrysosporium* were annotated, including multicopper oxidase 3B (*mco*3B), multicopper oxidase 3B-I5 (*mco*3B-I5), multicopper oxidase 3B-I10 (*mco*3B-I10), multicopper oxidase 2A (*mco*2A), and multicopper oxidase 4A (mco4A). After ARTP mutagenesis, the expression of *mco*3B and *mco*2A was significantly upregulated. Previous studies have shown that *mco*3 and *mco*2 are close together, separated by an intergenic region of only 0.8 kB, and both have transcriptional activity [9]. The expression levels of *mco*3B and *mco*2A in the Tx426 mutant strain were respectively 2.03 and 2.33 times higher than in the original strain, a more significant improvement than the 1.14- and 1.33-fold increases observed in Wx089 (Fig. 5D). This was consistent with the relative increases of laccase production in these two mutant strains. Therefore, positive mutations in *mco*3B and *mco*2A may be the reason for the significant increase of laccase activity after mutagenesis. Additionally, the expression of genes encoding lignin peroxidases (LiP) and manganese peroxidases (MnP) was also activated. The significant upregulation of LiP and MnP-related genes indicates that the two mutant strains may have a stronger lignin degradation ability. This further emphasizes the importance of changes in the expression of specific genes in metabolic pathways for biosynthetic and catabolic processes.

Based on DEG annotations from the eggNOG, GO, and KEGG databases, it stands to reason that the significant upregulation of the multicopper oxidase genes *mco*3B and *mco*2A in *P. chrysosporium* may be a major reason for the substantial increase of laccase activity after mutagenesis. This inference is consistent with previous findings, suggesting that regulating specific genes in metabolic engineering is important for improving the yield of target products. Future studies can further explore the specific mechanisms of these gene expression changes and their roles in metabolic pathways to provide a more in-depth theoretical basis for optimizing biosynthetic pathways.

## Conclusion

Laccases from white rot fungi have a wide range of practical applications. However, the mechanism of laccase secretion remains unclear, and the production capacity of current strains needs further improvement. This study focused on the white-rot fungus *P. chrysosporium* BZ103, which exhibits a remarkable dye decolorization ability. Experiments confirmed that this decolorization ability is related to the laccase secreted by the fungus. RT-PCR revealed that the expression pattern of the multicopper oxidase gene Mco3b was consistent with changes in enzyme activity in the presence of different dyes. Preliminary inferences suggest that this multicopper oxidase gene may encode the main laccase of the strain.

Further ARTP mutagenesis yielded the two mutant strains Tx426 and Wx089, which exhibited stable high laccase production, respectively 1.45 and 1.3 times that of the original strain. Transcriptome analysis showed that the multicopper oxidase genes *mco3B* and *mco2A* were significantly upregulated after mutagenesis, with expression levels of 2.03 and 2.33 times (Tx426 vs. CK) and 1.14 and 1.33 times (Wx089 vs. CK) those of the original strain, respectively. This may be a major reason for the significant increase of laccase activity after mutagenesis, providing a research direction for further genetically engineering laccase-hyperproducing white-rot fungal strains.

## Supplemental Materials

Supplementary data for this paper are available on-line only at http://jmb.or.kr.



## Figures and Tables

**Table 1:** Decolorization of dyes at different times with BZ103.

Synthetic dyes	Decolorization rate (%)			
	3	6	9	12
Phenol red	28.49 ± 1.25 c	39.1 ± 1.47 b	40.77 ± 1.51 ab	42.44 ± 1.31 a
Congo red	96.2 ± 0.5 b	96.69 ± 0.29 ab	96.86 ± 0.23 a	97.02 ± 0.06 a
Coomassie Bright Blue	93.59 ± 0.45 c	95 ± 0.3 b	95.81 ± 0.52 a	96 ± 0.25 a
Crystal violet	12.44 ± 2.51 c	21.98 ± 2.07 b	24.83 ± 1.64 ab	26.02 ± 1.86 a
Indigo carmine	89.87 ± 1.14 b	94.52 ± 0.5 a	95.33 ± 1.31 a	95.61 ± 0.96 a
Evans Blue	94.15 ± 0.93 b	97.87 ± 1.28 a	98.15 ± 0.9 a	99.08 ± 0.27 a
Azure A	10.58 ± 1.93 b	18.53 ± 3.09 a	21.44 ± 2.79 a	22.99 ± 2.66 a

**Table 2:** Laccase activity of BZ103 cocultured with dye at different time.

Synthetic dyes	Laccase activity (U/L)			
	3 d	6 d	9 d	12 d
Phenol Red	233.06 ± 19.01 c	444.9 ± 32.75 a	369.42 ± 17 b	269.46 ± 11.71 c
Congo Red	667.22 ± 37.04 c	886.01 ± 14.01 a	738.99 ± 25.63 b	695.41 ± 10.38 bc
Coomassie Bright Blue	811.73 ± 44.13 bc	923.69 ± 23.7 a	853.33 ± 47.58 b	746.41 ± 14.17 c
Crystal Violet	164.18 ± 10.28 c	305.79 ± 8.9 a	232.69 ± 20.71 b	185.87 ± 12.98 c
Indigo Carmine	443.55 ± 10.51 d	715.85 ± 24.45 a	635.73 ± 28.39 b	555.95 ± 29.27 c
Evans Blue	601.11 ± 41.81 c	745.27 ± 21.16 a	695.8 ± 5.63 b	610.88 ± 13.97 c
Azure A	127.5 ± 18.33 b	235.36 ± 28.7 a	213.29 ± 12.6 a	155.06 ± 31.78 b
